# 2013 Update on Celiac Disease and Eosinophilic Esophagitis

**DOI:** 10.3390/nu5093329

**Published:** 2013-08-22

**Authors:** Rinaldo Pellicano, Claudio De Angelis, Davide Giuseppe Ribaldone, Sharmila Fagoonee, Marco Astegiano

**Affiliations:** 1 Department of Gastro-Hepatology, Molinette Hospital, C.so Bramante 88, 10126 Turin, Italy; E-Mails: eusdeang@hotmail.com (C.D.); davrib_1998@yahoo.com (D.G.R.); mastegiano@cittadellasalute.to.it (M.A); 2 Molecular Biotechnology Center, University of Turin, 10126 Turin, Italy; E-Mail: sharmila.fagoonee@unito.it

**Keywords:** celiac disease, eosinophilic esophagitis, food allergy, autoimmune disorders

## Abstract

Celiac disease is a chronic, immune-mediated disorder, characterized by small intestinal inflammation and villous atrophy after the ingestion of gluten by genetically susceptible individuals. Several extraintestinal manifestations have been associated to celiac disease. Eosinophilic esophagitis is a primary disorder of the esophagus characterized by upper gastrointestinal symptoms, absence of gastroesophageal reflux disease and more than 15 eosinophils per high-power field in biopsy specimens. Both celiac disease and eosinophilic esophagitis are caused by aberrant, but distinct, immune responses to ingested antigens and can be responsive to restricted food intake. The aim of this review is to assess whether there is an association between these two pathologies. In the majority of the studies examined, including the studies in pediatric population, the prevalence of eosinophilic esophagitis in subjects with celiac disease was about 10-times that of the general population. We suggest searching for eosinophilic esophagitis in all children undergoing endoscopy for suspicious celiac disease.

## 1. Introduction

Celiac disease is a chronic, immune-mediated disorder, characterized by malabsorption of nutrients after the ingestion of wheat gluten or related proteins from rye and barley by genetically susceptible individuals expressing the human leukocyte antigen (HLA) class II molecules DQ2 or DQ8 [1] resulting in villus atrophy of the small intestinal mucosa. Prompt clinical and histologic improvement is observed following strict adherence to a gluten-free diet, and clinical and histologic relapse occurs when gluten is reintroduced [2]. Several extraintestinal manifestations, including anemia, osteopenia, neurologic symptoms, menstrual abnormalities, infertility, recurrent spontaneous abortions, growth retardation, dermatitis herpetiformis, aphthous stomatitis, dental defects, have been associated with celiac disease [3].

**Figure 1:**
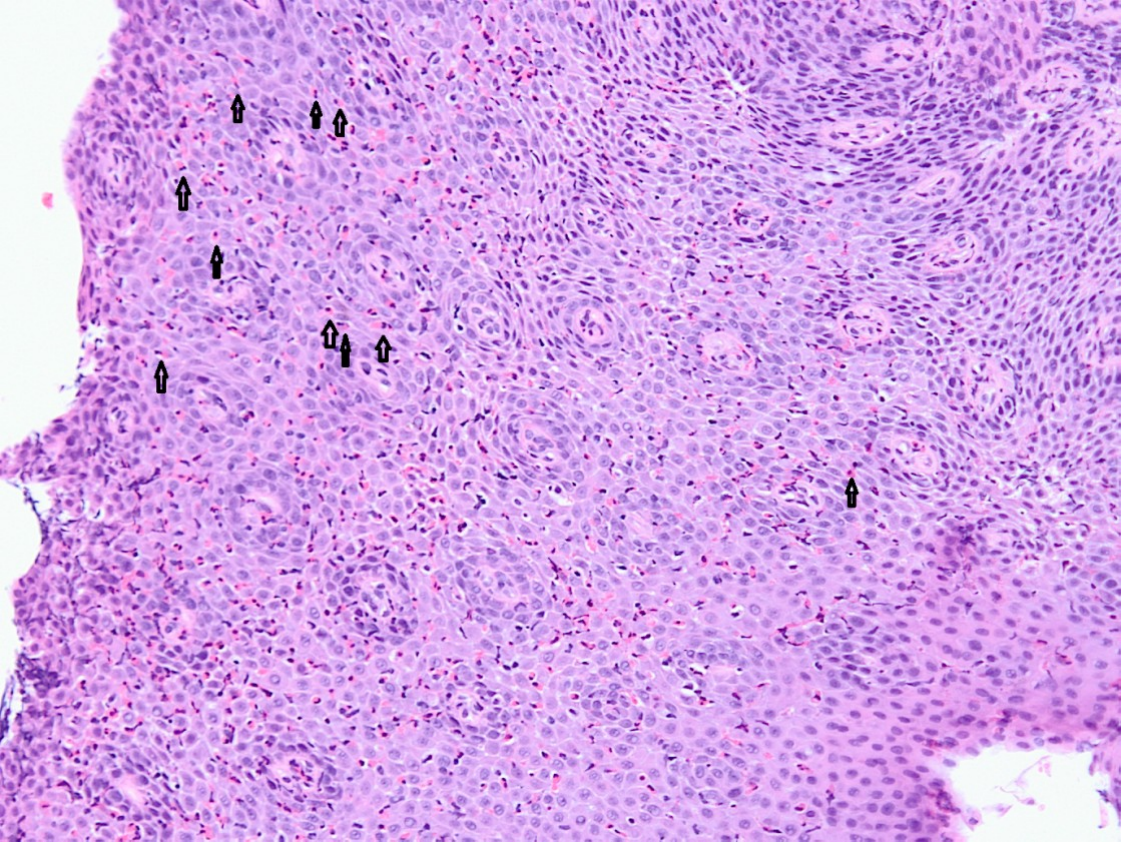
Classic histological findings of eosinophilic esophagitis: hypereosinophilia, usually with >15–20 eosinophils per high-powered field. Eosinophils in the squamous mucosa are visible (arrows). (Courtesy of Dr. Ezio David, MD, Molinette Hospital, Turin, Italy.)

Eosinophilic esophagitis was first described in 1978 [4]; however, it became recognized as a distinct clinical entity in 1995 [5]. It is a chronic inflammatory primary disorder of the esophagus, presenting with dysphagia and symptoms mimicking those of gastroesophageal reflux disease, including vomiting, regurgitation, nausea and epigastric pain. This disorder is characterized by esophageal mucosal biopsy containing more than 15 eosinophils per high-power field (Figure 1) and absence of gastroesophageal reflux disease, as shown by normal pH monitoring or lack of response to high-dose proton pump inhibitory therapy [6].

There are some classic endoscopic features including adherent whitish plaques, esophageal concentric rings, linear furrowing, but the esophagus can appear only slightly altered in some patients [7]. The squamous epithelium of the esophagus is normally devoid of eosinophils, but various disorders cause eosinophils to infiltrate the esophageal epithelium: parasitic infections, autoimmune disease, vasculitis, medications, gastroesophageal reflux disease [8]. When first described, eosinophilic esophagitis was believed to be a predominantly pediatric condition; however, it is now commonly diagnosed in adults as well as in children [9]. The clinical presentation of eosinophilic esophagitis may vary depending on age: younger children, generally present with non-specific symptoms of gastroesophageal reflux disease, abdominal pain or failure to thrive [10]; older children often present with dysphagia and esophageal food impaction [11]; however, asymptomatic low-grade counts of epithelial eosinophils (less than 15 eosinophils per high-power field) may be more common than has been estimated, but are of uncertain clinical significance [12]. A subset of patients with eosinophilic esophagitis responds to acid suppressive therapy, indicating some overlap between eosinophilic esophagitis and gastroesophageal reflux disease [6]; this is now considered as a separate entity labelled “proton pump inhibitors (PPI) responsive oesophageal eosinophilia” [13] In children, eosinophilic esophagitis has been shown to be associated with IgE- and non-IgE-mediated food allergy, and the majority of cases respond to elemental diets or specific food protein elimination [6]. By contrast, in adults the response to dietary interventions is less predictable, and the treatment more commonly relies on swallowed corticosteroid aerosols [6]. The poor response rate to dietary interventions in adults may be due to a lower prevalence of food allergy, and sensitization to inhalant allergens may play a more significant etiological role [6]. The data about the incidence of eosinophilic esophagitis range from 0.5 cases per 10,000 [14] to 1 in 10,000 in Ohio, USA [15] and the prevalence range from 0.89/10,000 in Western Australia [16] to four cases per 10,000 in Ohio, USA [15] and 5.5 cases per 10,000 in Olmsted County, Minnesota, USA [17]. The incidence seems to be increasing in both adults and children, though it is as yet unclear whether this is solely attributable to increasing awareness and detection of the disease or whether it represents a genuine phenomenon [18]. There is a male predominance, with 76% of adult and 66% of pediatric cases being diagnosed in males [6]. Since both celiac disease and eosinophilic esophagitis are caused by aberrant, but distinct, immune responses to ingested antigens and can be responsive to food elimination diets, the objective of our study was to verify if there is an association between these two conditions.

## 2. Experimental Section

Articles regarding the association of these two diseases were identified through MEDLINE search using the terms “celiac disease or celiac sprue or gluten AND eosinophilic esophagitis”. The search was also performed using reference lists from published articles. The titles of these publications and their abstracts were scanned in order to eliminate duplicates and irrelevant articles. The final date of the MEDLINE search was June 19, 2013.

## 3. Results

The search identified 30 publications (from November 2001 to May 2013) on this subject. We read the abstracts of all articles and selected the 13 original articles in which associations between the two diseases were addressed; three were excluded because there were no data about the prevalence of the diseases. Celiac disease and eosinophilic esophagitis have been described in the same patient for the first time in a case report of 2007 [19] in a 7-year old black male with reactive airway disease, eczema and type 1 diabetes mellitus referred to the gastroenterology clinic for positive celiac serologic findings in recurrent abdominal pain. The eosinophilic esophagitis responded to an elimination diet with normalization of esophageal histology and subsequently recurred with reintroduction of cow’s milk protein (while celiac disease’s histology remained in remission with gluten-free diet). A second report of three associated cases was described in the same year [20] with a reported prevalence of eosinophilic esophagitis in patients with celiac disease of 9%, nine times higher than that expected in the general population (1:100) [21]. In one patient eosinophilic esophagitis disappeared after gluten-free diet; in the other two cases, the gluten-free diet did not have any effect on the eosinophilic infiltrate, but both subjects were not compliant to the gluten-free diet. Another study [22] reported a 35% of prevalence of celiac disease in 17 patients affected by eosinophilic esophagitis investigated for upper gastrointestinal symptoms, with a significant clinical and histological remission on gluten-free diet compared to the group of patients with eosinophilic esophagitis without celiac disease. In an Australian study regarding seven children with eosinophilic esophagitis and celiac disease [23], no patients with food allergy were reported; the prevalence of eosinophilic esophagitis in the cohort of patients with celiac disease was 3.1%; two of seven patients who underwent repeated endoscopic examinations showed improved duodenal histology but persistent eosinophilic esophagitis on gluten-free diet. In another Australian study published in 2010 [24], the prevalence of esophageal eosinophilia in children with celiac disease who had concurrent esophageal biopsies was 8.2% (10 of 121), 60% males, 30% had normal-appearing esophageal mucosa at endoscopy; children who had undergone repeated endoscopic examinations showed recovery of duodenal mucosa but no resolution of esophageal eosinophilia on a gluten-free diet alone. The association of celiac and eosinophilic esophagitis may not be a true association but a matter of biased enrollment in the above studies: regarding HLA DQ2 and/or DQ8, a study [25] showed that these alleles were not present in eosinophilic esophagitis at a greater rate than in healthy controls. But a recent study [26] demonstrated, a clear association between celiac disease and eosinophilic esophagitis in both pediatric and adult populations: the standardized incidence ratio of eosinophilic esophagitis in patients with celiac disease was 16.0 (95% CI, 8.7–25.5). A general population-based study on adults [27] did not find any association between eosinophilic esophagitis and celiac disease, whereas the latest one [28] showed a prevalence of 1.2% of eosinophilic esophagitis in children with celiac disease (Table 1).

**Table 1:** Published study about prevalence of eosinophilic esophagitis (EoE) in patients affected by celiac disease (CD).

Study	Country	Prevalence of EoE in CD population	% of Pediatric patients	% of Male in EoE patients	Population
A [22]	Australia	3.1% (7 of 221)	100	43	Tertiary center
B [23]	Australia	8.2% (10 of 121)	100	60	Tertiary center
C [25]	USA	0.97% (14 of 1439)	20.6	57	Tertiary center
D [27]	Canada	1.2% (3 of 245)	100	100	General population

## 4. Discussion

Both celiac disease and eosinophilic esophagitis are distinct clinical entities except for a few minor similarities. Celiac disease is a Th1 mediated disorder which aligns with autoimmunity, triggered by the ingestion of food containing gluten and affects females:males at a ratio of 2:1 [29]. By contrast, eosinophilic esophagitis has been shown to be a Th2-mediated disorder, which is triggered by exposure to dietary allergens causing infiltration of the esophageal mucosa by T lymphocytes, mast cells and eosinophils and predominates in males, with a 3:1 ratio to females. There is also an overexpression of eotaxin-3 and interleukin-5 [6] in the latter. Affliction of 8% of first-degree relatives of patients with celiac disease is similar to that reported in 10% of first-degree relatives of patients with eosinophilic esophagitis [30]. The genetic basis for celiac disease (*i.e.*, HLA DQ2) is well established and differs from that of eosinophilic esophagitis [25] whose etiology is far from clear, but in nearly 50% of the cases, it is associated with an allergy to food or to aeroallergens [15]. In children, IgE-dependent mechanism for eosinophilic esophagitis is supported; for instance, it was shown that affected patients have IgE sensitization to a wide variety of foods, although not all patients had evidence of food-specific IgE [31]. In one study, a higher level of IgE sensitization to food allergens was observed in patients with eosinophilic esophagitis alone compared to patients with both pathologies, and this led the authors to hypothesize that patients with both pathologies have elevated esophageal eosinophils for reasons different from allergy, with a significant clinical and histological remission on gluten-free diet [22]. Increased intestinal mucosal permeability secondary to celiac disease has been suggested as a contributing factor in the development of atopy [32,33]. Damaged intestinal barrier may expose the local intestinal immune system to macromolecules and lead to transport of these undigested proteins to other body sites, hence facilitating development of hypersensitivity reactions in a predisposed individual in and away from the gastrointestinal tract [23]. By contrast, in adults, the response to dietary interventions is less predictable, and treatment more commonly relies on swallowed corticosteroid aerosols [6]. The clinical significance of eosinophilic esophagitis as an incidental finding is uncertain; if the main treatment goal is suppression of clinical symptoms, asymptomatic eosinophilic esophagitis may not require any therapy, but as the natural history of eosinophilic esophagitis in largely unknown, it is unclear what proportion is at risk of developing esophageal strictures and dysphagia in the long term [34].

## 5. Conclusions

In summary, although there are fundamental differences in the pathophysiological mechanisms involved in eosinophilic esophagitis and celiac disease, these conditions may coexist and the prevalence is higher than anticipated. Our review highlights the importance of obtaining routine esophageal biopsies in children undergoing endoscopy for diagnosis of celiac disease irrespective of whether the esophagus appears normal or abnormal at endoscopy; however, asymptomatic low-grade counts of epithelial eosinophils are of uncertain clinical significance.
